# Lulworthinone, a New Dimeric Naphthopyrone From a Marine Fungus in the Family Lulworthiaceae With Antibacterial Activity Against Clinical Methicillin-Resistant *Staphylococcus aureus* Isolates

**DOI:** 10.3389/fmicb.2021.730740

**Published:** 2021-10-01

**Authors:** Marte Jenssen, Philip Rainsford, Eric Juskewitz, Jeanette H. Andersen, Espen H. Hansen, Johan Isaksson, Teppo Rämä, Kine Ø. Hansen

**Affiliations:** ^1^Marbio, The Norwegian College of Fishery Science, Faculty of Biosciences, Fisheries and Economics, UiT the Arctic University of Norway, Tromsø, Norway; ^2^Department of Chemistry, Faculty of Science and Technology, UiT the Arctic University of Norway, Tromsø, Norway; ^3^Research Group for Host Microbe Interactions, Department of Medical Biology, Faculty of Health Sciences, UiT the Arctic University of Norway, Tromsø, Norway

**Keywords:** antibacterial, marine fungi *sensu stricto*, Lulworthiales, lulworthinone, MRSA, natural product, mycology, natural product artifact

## Abstract

The emergence of drug-resistant bacteria is increasing rapidly in all parts of the world, and the need for new antibiotics is urgent. In our continuous search for new antimicrobial molecules from under-investigated Arctic marine microorganisms, a marine fungus belonging to the family Lulworthiaceae (Lulworthiales, Sordariomycetes, and Ascomycota) was studied. The fungus was isolated from driftwood, cultivated in liquid medium, and studied for its potential for producing antibacterial compounds. Through bioactivity-guided isolation, a novel sulfated biarylic naphtho-α-pyrone dimer was isolated, and its structure was elucidated by spectroscopic methods, including 1D and 2D NMR and HRMS. The compound, named lulworthinone (**1**), showed antibacterial activity against reference strains of *Staphylococcus aureus* and *Streptococcus agalactiae*, as well as several clinical MRSA isolates with MICs in the 1.56–6.25 μg/ml range. The compound also had antiproliferative activity against human melanoma, hepatocellular carcinoma, and non-malignant lung fibroblast cell lines, with IC_50_ values of 15.5, 27, and 32 μg/ml, respectively. Inhibition of bacterial biofilm formation was observed, but no eradication of established biofilm could be detected. No antifungal activity was observed against *Candida albicans*. During the isolation of **1**, the compound was observed to convert into a structural isomer, **2**, under acidic conditions. As **1** and **2** have high structural similarity, NMR data acquired for **2** were used to aid in the structure elucidation of **1**. To the best of our knowledge, lulworthinone (**1**) represents the first new bioactive secondary metabolite isolated from the marine fungal order Lulworthiales.

## Introduction

Antimicrobial resistance is quickly developing as a worldwide threat, causing problems not only in the general community but also in healthcare facilities. Infections caused by methicillin-resistant *Staphylococcus aureus* (MRSA) has become a worldwide health menace (WHO, 2014). There is an urgent need to develop new antibiotics to fight these resistant microbes. The fungal kingdom has historically played an important role in the discovery and development of antibiotics and other drugs against non-infective diseases (Demain, 2014). The penicillins and cephalosporins are examples of important antibiotics isolated from fungi (Demain, 2014), from the genera *Penicillium* and *Sarocladium* (one syn. *Cephalosporium*), respectively. In marine natural product discovery, the genera *Aspergillus* and *Penicillium* have proven to be the most prolific producers of new compounds with biological activities (Imhoff, 2016). As the focus of marine natural product discovery has been on mold fungi belonging to the few genera mentioned above, the strictly marine clades of fungi remain understudied (Overy et al., 2014).

One of the understudied marine clades include the fungal order Lulworthiales from which no secondary metabolites have been reported since the discovery of the type genus and species, *Lulworthia fucicola*, in the beginning of the twentieth century (Sutherland, 1915). The order Lulworthiales was established in 2000 to accommodate the new family Lulworthiaceae in the class Sordariomycetes (Kohlmeyer et al., 2000). More recently, a new subclass, Lulworthiomycetidae, was described containing the orders Lulworthiales and Koralionastetales (Maharachchikumbura et al., 2015). Lulworthiaceae is the sole family in the Lulworthiales order, and Lulworthiaceae spp. are regarded as strictly marine species, which include the following genera: *Cumulospora*, *Halazoon*, *Hydea*, *Kohlmeyerella*, *Lulwoana*, *Lulworthia*, *Lindra*, *Matsusporium*, and *Moleospora* (Poli et al., 2020). Recently, a novel genus was introduced to the Lulworthiaceae, *Paralulworthia*, with two new species described, *Paralulworthia gigaspora* and *Paralulworthia posidoniae* (Poli et al., 2020). Hyde et al. (2020) also included the following genera in the family: *Haloguignardia*, *Lolwoidea*, *Moromyces*, *Orbimyces*, *Rostrupiella*, and *Sammeyersia*.

Fungi in the family Lulworthiaceae have been isolated from a variety of substrates and environments. Some examples include corals (Góes-Neto et al., 2020), plants located in salt marches (Calado et al., 2019), seagrass (Poli et al., 2020), Portuguese marinas (Azevedo et al., 2017), sandy beaches of the Cozumel island in Mexico (Velez et al., 2015), brown seaweed (Zuccaro et al., 2008), and driftwood (Rämä et al., 2014). The distribution of Lulworthiales fungi in marine habitats has been studied throughout the history of marine mycology (Johnson, 1958; Kohlmeyer et al., 2000; Koch et al., 2007; Rämä et al., 2014; Azevedo et al., 2017; Góes-Neto et al., 2020), but the biosynthetic potential of these fungi has not been investigated, most likely due to the special knowledge required for their isolation (Overy et al., 2019) and low growth rates.

In this paper, we report the isolation of a new antibacterial compound, lulworthinone (**1**), from a liquid culture of a marine fungus belonging to Lulworthiaceae (isolate 067bN1.2). We elucidate the structure of **1** and study its bioactivity against prokaryotic and eukaryotic cells with focus on antibacterial activity against clinical MRSA isolates. Compound **1** represents the first secondary metabolite reported from this order of fungi, and to the best of our knowledge, the first biarylic dimeric naphtho-α-pyrone substituted with a sulfate group. Initially, the compound was isolated using preparative HPLC under acidic conditions. As this procedure caused significant wear and tear to the equipment, the isolation was switched to flash chromatography under neutral conditions. When comparing spectroscopic data from the two samples, one isolated at neutral and one at acidic conditions, structural differences were observed. It was later determined that **1** concerts into the artifact **2** under acidic conditions.

## Materials and Methods

### Biological Material and Phylogenetic Analysis of Isolate 067bN1.2

The marine fungus 067bN1.2 was isolated from a dead pine (*Pinus* sp.) collected in the splash zone in Kongsfjord, Berlevåg Norway in 2010. The isolate grew from a small wooden cube plated onto agar medium (specified below) during a campaign to study wood-inhabiting fungi of 50 intertidal and sea-floor logs along the Northern Norwegian coast, where Lulworthiales was one of the five most frequent orders isolated (Rämä et al., 2014). The fungus was subcultured and DNA sequenced, and the fungus was phylogenetically placed in the Lulworthiales order (isolate TR498 represents 067bN1.2 in Rämä et al., 2014). At the time of the publication (2014), the closest match from Blast, based on a 5.8S/large ribosomal subunit (LSU) dataset, was *Lulworthia medusa* (LSU sequence: AF195637). The following primer pairs were used for the internal transcribed spacer (ITS), LSU and small ribosomal subunit (SSU) sequencing, respectively: ITS5-ITS4 (White et al., 1990), LR0R-LR5 (Vilgalys and Hester, 1990; Rehner and Samuels, 1994), and NS1-NS4 (White et al., 1990). The ITS, LSU, and SSU sequences are deposited in GenBank under the following accessions: MW377595, MW375591, and MW375590. The mycelium of the fungus was preserved on pieces of agar in 20% glycerol solution at −80°C.

To identify the isolate 067bN1.2 growing as an asexual morph in culture and determine its systematic position within the order Lulworthiales, a phylogenetic analysis was run using a dataset consisting of nrSSU, nrITS, and nrLSU sequences. The reference sequences included in the analyses were sampled based on recent phylogenetic studies focusing on Lulworthiales (Azevedo et al., 2017; Poli et al., 2020) and retrieved from Genbank (Supplementary Table 1). Sequences for each gene were aligned individually using the E-INS-I and G-INS-I algorithms of MAFFT v7.388 (Katoh et al., 2002; Katoh and Standley, 2013) in Geneious Prime v.11.0.4 followed by manual adjustment. The concatenated dataset consisting of SSU, 5.8S, and LSU sequences and having a length of 2,270 nt was run through PartitionFinder v2.1.1 (Lanfear et al., 2017) to test for best-fit partitioning schemes and evolutionary models with the following settings: models MrBayes, linked branch lengths, greedy search, and AIC and BIC model selection (Lanfear et al., 2012). This suggested three partitions with varying models: symmetrical model with equal base frequencies and gamma distributed rate variation among sites without (SYM+G) and with (SYM+I+G) invariable sites and general time reversible model with variable base frequencies and gamma distributed rate variation among sites (GTR+G). A phylogenetic analysis was set up applying suggested models using Parallel-MPI MrBayes v3.2.7a with beagle, and was run for 5,000,000 generations or until average standard deviation of split frequencies was below 0.0009 with sampling each of the 2,500 generations (Ronquist et al., 2012). In addition, RAxML in Geneious v10.2.3 was run with the same partitions under GTRCAT and GTRGAMMA using rapid-bootstrapping algorithm with 2,000 replicates with search for best scoring ML tree (Stamatakis, 2006). The resulting MrBayes tree was similar to the RAxML tree, excluding some of the basal nodes within Lulworthiaceae shown as polytomies in the MrBayes tree.

### Fungal Cultivation and Extraction

For the purpose of this study, the fungal isolate was plated from glycerol stock and grown on nutrient-poor malt agar with sea salts [4 g/L malt extract (Moss Malt Extrakt, Jensen & Co AS), 40 g/L sea salts (S9883, Sigma-Aldrich), 15 g/L agar (A1296, Sigma-Aldrich) and Milli-Q^®^ H_2_O] until the growth covered the entire agar plate (approximately 40 days). Milli-Q^®^ H_2_O was produced with the in-house Milli-Q^®^ system. One-half of the agar plate covered in mycelium was used to inoculate each liquid culture, in malt medium with added sea salts (4 g/L malt extract, 40 g/L sea salts). Two cultures of 200 ml were inoculated and incubated for 107 days at static conditions and 13°C. Before the addition of resin for extraction, mycelium was taken from the culture for inoculation of another round of cultures. The second cultivation contained four cultures with 250 ml of malt extract medium supplemented with sea salts and cultivated under the same conditions for 83 days. The total culture volume used for the extraction of **1** was 1.4 L. The cultures were extracted using Diaion HP-20 resin (13607, Supelco) and methanol (20864, HPLC grade, VWR) as described previously (Kristoffersen et al., 2018; Schneider et al., 2020). The extract was dried in a rotary evaporator at 40°C under reduced pressure and stored at −20°C.

### Dereplication

As part of our ongoing search for antimicrobial compounds, extracts of marine microorganisms are fractioned into six fractions using flash chromatography, as previously described (Schneider et al., 2020). When we investigated the antibacterial potential of fractions produced from several understudied marine fungi, one fraction from isolate 067bN1.2 piqued our interest due to its antibacterial activity. In the active fraction, **1** was the dominating peak. The monoisotopic mass, calculated elemental composition and fragmentation pattern of **1** was determined using UHPLC-ESI-HRMS. UHPLC-ESI-HRMS was performed with positive ionization mode, using an Acquity I-class UPLC with an Acquity UPLC C18 column (1.7 μm, 2.1 mm × 100 mm), coupled to a PDA detector and a Vion IMS QToF (all from Waters). Compounds were eluted with a gradient over 12 min, from 10 to 90% acetonitrile (LiChrosolv, 1.00029, Supelco) with 0.1% formic acid (Sigma-Aldrich) in Milli-Q H_2_O and a flow rate of 0.45 ml/min. Waters UNIFI 1.9.4 Scientific Information System was used to process and analyze the data. Elemental compositions of compounds in the samples were used to search relevant databases, such as Chemspider, in order to identify known compounds. Since the calculated elemental composition gave no hits in database searches, **1** was nominated for isolation.

### Isolation of 1

Initial attempts to isolate **1** was performed using mass guided preparative HPLC. This strategy proved difficult due to extensive binding of the compound to an Atlantis Prep C18 (10 μM, 10 × 250 mm) (Waters) column, leading to inefficient isolation and column contamination. The preparative system and mobile phases used were as previously described (Schneider et al., 2020). The resulting sample (referred to as compound **2**) was later used to assist in structure elucidation of compound **1**.

To avoid wear and tear of the preparative HPLC system, attempts were made to isolate **1** using flash chromatography. The dried extract was dissolved in 90% methanol, and 2 g of Diaion HP-20SS (13615, Supelco) was added before removing the solvent under reduced pressure. Flash columns were prepared as previously described (Kristoffersen et al., 2018). The column was equilibrated using 5% methanol, before the dried extract-Diaion HP-20SS mixture was applied to the top of the column (maximum 2 g of extract per round). The fractionation was performed on a Biotage SP4^TM^ system (Biotage) with a flow rate of 12 ml/min and a stepwise gradient from 5 to 100% methanol over 32 min. The following stepwise elution method was used: methanol:water (5:95, 25:75, 50:50, 75:25, 6 min per step, resulting in 12 fractions) followed by methanol (100% over 12 min, resulting in six fractions). The MeOH fractions were analyzed using UHPLC-ESI-HRMS. In the second fraction eluting at 100% MeOH, **1** was the dominating peak and was submitted for NMR and bioactivity analysis. The sample of **1** was therefore produced by pooling the second fraction eluting at 100% MeOH from multiple rounds of flash fractionation and drying the resulting volume under reduced pressure.

### Structure Elucidation of 1

The structure of **1** was established by 1D and 2D NMR experiments. NMR spectra were acquired in DMSO-*d*_6_ and methanol-*d*_3_ on a Bruker Avance III HD spectrometer operating at 600 MHz for protons, equipped with an inverse TCI probe cryogenically enhanced for ^1^H, ^13^C, and ^2^H. All NMR spectra were acquired at 298 K, in 3-mm solvent matched Shigemi tubes using standard pulse programs for proton, carbon, HSQC, HMBC, HMQC (*J* = 4–5 Hz), COSY, NOESY, ROESY and 1,1-ADEQUATE experiments with gradient selection and adiabatic versions where applicable. ^1^H/^13^C chemical shifts were referenced to the residual solvent peak (δ_*H*_ = 2.50 PPM, δ_*C*_ = 39.52 PPM for DMSO). All data were acquired and processed using Topspin 3.5pl7 (Bruker Biospin) including the structure elucidation module CMC-se v. 2.5.1. ^13^C prediction was done using Mestrelabs MestReNova software version 14.2.0-26256 with the Modgraph NMRPredict Desktop. Optical rotation data were obtained using an AA-10R automatic polarimeter (Optical Activity LTD).

Lulworthinone (**1**): green colored film. [α]^20^_*D*_ -120 ± 0.02 (*c* 0.2 DMSO). ^1^H and ^13^C NMR spectroscopic data, Supplementary Table 3. HRESIMS *m/z* 741.2204 [M+H]^+^ (calculated for C_3__7_H_4__1_O_1__4_S, 741.2217).

### Minimal Inhibitory Concentration Determination Against Reference Bacteria

The Minimal Inhibitory Concentration (MIC) of **1** against a panel of Gram-positive and Gram-negative reference bacteria was determined by broth microdilution, at final concentrations 0.2–100 μg/ml (twofold dilution series). The experiments were performed with three technical replicates. The panel of reference bacteria consisted of the following strains: *S. aureus* (ATCC 25923), MRSA (ATCC 33591), *Escherichia coli* (ATCC 25922), *Pseudomonas aeruginosa* (ATCC 27853), *Enterococcus faecalis* (ATCC 29212), and *Streptococcus agalactiae* (ATCC 12386), all strains from LGC Standards (Teddington). Briefly, the bacteria were inoculated from freeze stock onto blood agar plates (University Hospital of North Norway) and transferred to liquid medium for overnight incubation at 37°C. *S. aureus*, *E. coli*, and *P. aeruginosa* were grown in Brain Heart Infusion medium (BHI, 53286, Sigma-Aldrich), and *E. faecalis* and *S. agalactiae* were grown in Difco^TM^ Mueller Hinton medium (MH, 275730, BD Biosciences). After overnight incubation in the respective media, the bacteria were brought to exponential growth by addition of fresh media, and incubated to reach a turbidity of 0.5 McFarland standard. The bacteria were diluted in their respective media 1:1,000 prior to addition. Subsequently, the bacteria were added to 96-well microtiter plates at 50 μl/well. A mixture of 50 μl of autoclaved Milli-Q^®^ H_2_O and 50 μl fresh autoclaved media was used as negative control, and 50 μl of autoclaved Milli-Q^®^ H_2_O was added to 50 μl of bacteria suspension as growth control. The compound was diluted in DMSO and autoclaved Milli-Q^®^ H_2_O (highest concentration of DMSO in the assay was 0.5%), and 50 μl was added to the bacterial suspension. Final volume in the wells was 100 μl. The plates were incubated overnight at 37°C. After incubation, growth was measured by absorbance at 600 nm with 1420 Multilabel Counter VICTOR^3^^TM^ (Perkin Elmer). Assay controls with gentamicin in a dilution series are routinely run, as well as routine counting of CFUs for each bacterium. For the strains where the compound displayed activity, the MIC was determined with three biological replicates each containing three technical replicates (*n* = 9). The lowest concentration of **1** that completely inhibited the growth of the bacteria was determined as the MIC.

To investigate if **1** had a bacteriocidal or bacteriostatic effect on *S. aureus* and *S. agalactiae*, the compound was inoculated together with the bacteria, as described above, and after overnight incubation, the inoculum was plated onto agar and incubated overnight at 37°C. The experiment was done with 12.5 and 25 μg/ml concentrations of **1** in triplicate, with two biological replicates (*n* = 6). Inspired by Zheng et al. (2007), we tested **1**, together with reserpine (broad spectrum efflux pump inhibitor) against the Gram-negative reference strains *E. coli* and *P. aeruginosa*. The assay was conducted as described above, with reserpine (L03506, Thermo Fisher Scientific) added to a final concentration of 20 μg/ml.

### Minimal Inhibitory Concentration Determination Against Clinical Bacterial Isolates

Initial testing of **1** was conducted against a panel containing clinically relevant antibiotic-resistant bacteria: Gram-positive MRSA, vancomycin-resistant *Enterococcus faecium* (VRE), and Gram-negative bacteria resistant to extended-spectrum beta-lactamases as well as carbapenemases (ESBL-Carba) (detailed information about the clinical isolates can be found in Supplementary Table 2). The initial testing was conducted at one concentration, 100 μg/ml.

The final antibacterial testing of **1** was executed using the five clinical MRSA isolates and the VRE isolates (Supplementary Table 2). The isolates were tested by broth microdilution according to the Clinical Laboratory Standard Institute (CLSI) (2012) method MO7-A9. In brief, **1** was solubilized with 100% DMSO and diluted with autoclaved Milli-Q^®^ H_2_O to prepare a 200 μg/ml working solution. The final DMSO concentration did not exceed 1% to exclude any artificial influence on the assay. The bacterial inoculum was prepared to contain 1 × 10^6^ CFU/ml in cationic-adjusted BBL^TM^ Mueller-Hinton II broth (BD). The inoculum was mixed in a 1:1 ratio with the working solution of **1** (twofold dilutions, ranging from 0.2 to 100 μg/ml) for a final amount of 5 × 10^5^ CFU/ml in each well of a 96-well round-bottom polypropylene plate (Greiner Bio-One GmbH). Growth control (without compound) and sterility control (without bacteria) were included for each strain. Each strain was tested in three independent biological replicates with four technical replicates on consecutive days. As quality assurance for the assay, the protocol was also performed with *E. coli* ATCC 25922 using Gentamicin (Merck Life Science) as a reference antibiotic. The 96-well plates were incubated at 37°C for 24 h without shaking. The MIC values were defined as the lowest concentration of **1** resulting in no visual bacterial growth, determined by visual inspection and 600 nm absorbance measurements with CLARIOstar plate reader (BMG LABTECH).

### Inhibition of Biofilm Production and Eradication of Established Biofilm

Inhibition of biofilm production by **1** of *Staphylococcus epidermidis* (ATCC 35984, LGC Standards) was determined at final concentrations 0.2–100 μg/ml (twofold dilution series). Briefly, the bacteria were inoculated from freeze stock onto blood agar plates (University Hospital of North Norway) and transferred to tryptic soy broth (TSB, 22092, Sigma-Aldrich) for overnight incubation at 37°C. The overnight cultures were subsequently diluted 1:100 in fresh TSB with 1% glucose and added to 96-well microtiter plates, 50 μl/well. Positive control was *S. epidermidis* in fresh media with glucose, and negative control was a non-biofilm producing *Staphylococcus haemolyticus* (clinical isolate 8-7A, University Hospital of North Norway) in fresh media with glucose. The compound was diluted in DMSO and autoclaved Milli-Q^®^ H_2_O (highest concentration of DMSO in the assay was 0.5%), and 50 μl was added to the bacterial suspension. Final volume in the wells was 100 μl. The plates were incubated at 37°C overnight. Growth inhibition of the bacterium was determined by visual inspection of the plates prior to further treatment. The bacterial suspension was poured out and the biofilm was fixated by heat, before adding 70 μl of 0.1% crystal violet solution (V5265, Sigma-Aldrich) and staining for 5 min. The crystal violet solution was removed and the wells were washed with water before the plates were dried by heat. The bound crystal violet was dissolved in 70 μl of 70% ethanol, and the presence of violet color, indicating biofilm formation, was measured at 600 nm absorbance using a 1420 Multilabel Counter VICTOR^3^^TM^ reader. Percent biofilm formation was calculated using the equation below. The data were visualized using GraphPad Prism 8.4.2, and the built-in ROUT method was used to detect and remove outliers from the dataset (*Q* = 1%).


(1) $$\begin{array}{c} \text{Percent}\left(\%\right)\mathrm{biofilm}\mathrm{formation}\\ =\frac{\left(\mathrm{absorbance}\mathrm{treated}\mathrm{wells}-\mathrm{absorbance}\mathrm{negative}\mathrm{control}\right)}{\left(\mathrm{absorbance}\mathrm{positive}\mathrm{control}-\mathrm{absorbance}\mathrm{negative}\mathrm{control}\right)}\\ \text{×}100 \end{array}$$

To determine whether **1** could eradicate biofilm established by *S. epidermidis*, a modified biofilm inhibition assay protocol was performed. Here, the bacteria were grown overnight in a microtiter plate to allow the biofilm to be established prior to the addition of **1**. After addition of **1**, the plates are incubated overnight. Following this, the biofilm was fixated and colored and results were read as stated above. The experiment was conducted once with three technical replicates with concentrations of 0.2–100 μg/ml (twofold dilution series).

### Determination of Antiproliferative Activity Toward Human Cell Lines

The antiproliferative activities of **1** was evaluated against the melanoma cell line A2058 (ATCC, CRL-11147^TM^), the hepatocellular carcinoma cell line HepG2 (ATCC, HB-8065^TM^), and the non-malignant lung fibroblast cell line MRC5 (ATCC, CCL-171^TM^) in a MTS *in vitro* cell proliferation assay. The compound was tested in concentrations from 6.3 to 100 μg/ml against all cell lines, with three biological replicates each containing three technical replicates (*n* = 9). A2058 was cultured and assayed in Dulbecco’s Modified Eagle’s Medium (D-MEM, D6171, Sigma-Aldrich). HepG2 was cultured and assayed in MEM Earle’s (F0325, Biochrom) supplemented with 5 ml of non-essential amino acids (K0293, Biochrom) and 1 mM sodium pyruvate (L0473, Biochrom). MRC5 was cultured and assayed in MEM Eagle (M7278, Sigma-Aldrich) supplemented with 5 ml of non-essential amino acids, 1 mM sodium pyruvate, and 0.15% (w/v) sodium bicarbonate (L1713, Biochrom). In addition, all media were supplemented with 10% fetal bovine serum (FBS, S1810, Biowest), 10 μg/ml gentamicin (A2712, Biochrom), and 5 ml of glutamine stable (200 mM per 500 ml medium, X0551, Biowest). Briefly, the cells were seeded in 96-well microtiter plates (Nunclon Delta Surface, VWR) at 2,000 cells/well for A2058, 4,000 cells/well for MRC5, and 20,000 cells/well for HepG2. After incubation for 24 h in 5% CO_2_ at 37°C, the media was replaced and compound was added, generating a total volume of 100 μl/well. A2058 and MRC5 were incubated for 72 h before assaying, and HepG2 for 24 h. Subsequently, 10 μl of CellTiter 96 AQueous One Solution Reagent (G358B, Promega) was added to each well and the plates were incubated for 1 h at 37°C. Following this, the absorbance was measured at 485 nm with a DTX 880 multimode detector (Beckman Coulter). Negative controls were cells assayed with their respective cell media, and positive controls were cells treated with 10% DMSO (D4540, Sigma-Aldrich). Percent cell survival was calculated using the equation below. The data were visualized using GraphPad Prism 8.4.2 and IC_50_ was calculated. The built-in ROUT method was used to detect and remove outliers from the dataset (*Q* = 1%).


(2) $$\begin{array}{c} \text{Percent}\left(\%\right)\mathrm{cell}\mathrm{survival}:\\ \frac{\left(\mathrm{absorbance}\mathrm{treated}\mathrm{wells}-\mathrm{absorbance}\mathrm{positive}\mathrm{control}\right)}{\left(\mathrm{absorbance}\mathrm{negative}\mathrm{control}-\mathrm{absorbance}\mathrm{positive}\mathrm{control}\right)}\\ \times 100 \end{array}$$

### Minimal Inhibitory Concentration Determination Against *Candida albicans*

The MIC of **1** was determined by broth microdilution against *C. albicans* (ATCC 90028, LGC Standards), at final concentrations of 0.2–100 μg/ml (twofold dilution series). The experiment was performed as one biological replicate, with three technical replicates (*n* = 3). Briefly, the fungus was inoculated from freeze stock onto potato dextrose agar [24 g/L potato dextrose broth (P6685, Sigma-Aldrich), 15 g/L agar (A1296, Sigma-Aldrich)] and incubated overnight at 37°C. From the overnight culture, five to eight colonies were transferred to 5 ml of sterile 0.9% NaCl, before the cell density was adjusted to 1–5 × 10^6^ cells/ml by adding 0.9% NaCl. The cell density was evaluated with 0.5 McFarland standard (Remel 0.5 McFarland Equivalence Turbidity Standard, 10026732, Thermo Fisher Scientific). The fungal suspension was further diluted 1:50, and then 1:20 (1–5 × 10^3^ CFU/ml) in RPMI medium (R7755, Sigma-Aldrich) with 0.165 mol/L MOPS (M3183, Sigma-Aldrich) and 10.25 ml of L-glutamine. The compound was added to the microtiter plate together with the fungal suspension (1:1), to a final volume of 200 μl. The final concentration of fungal cells was 0.5–2.5 × 10^3^ CFU/ml. Absorbance in the wells was measured with 1420 Multilabel Counter VICTOR^3^^TM^ right after addition of compound, after 24 h and after 48 h. The plates were incubated at 37°C. Amphotericin B was used as negative control at final concentration 8 μg/ml. Growth control contained fungal suspension and autoclaved Milli-Q^®^ H_2_O.

## Results

### Systematic Placement of the Fungal Isolate 067bN1.2

**FIGURE 1:**
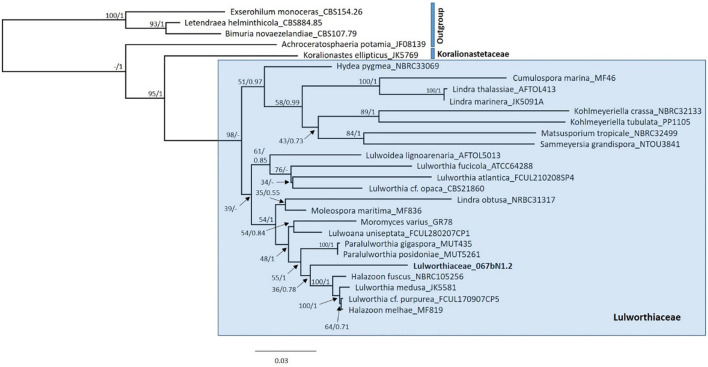
Maximum Likelihood tree (RAxML) from the combined analysis of 5.8S, SSU, and LSU from isolates of Lulworthiaceae. One isolate from Koralionastetaceae was included, and four strains as outgroups. Node support is given as Bootstrap support values at the nodes, and posterior probabilities are included where the branching was alike (BS/PP). The isolate under investigation, Lulwortihaceae_067bN1.2, is highlighted in bold. Due to topological similarity only the ML tree is shown here containing both Bayesian posterior probabilities and Bootstrap support values. Bayesian Inference tree can be found in Supplementary Figure 1. – indicates that the node is missing in the Bayesian analysis. No support value is given to the node separating the outgroup taxa from the ingroup in ML analysis.

Due to lack of distinct morphological characters of the cultured asexual morph and closely related reference sequences in GenBank, the fungus is identified to family level, as Lulworthiaceae sp., for the purpose of this study. A phylogenetic study was carried out with 28 taxa (including outgroups and isolate 067bN1.2), all representing different species, as shown in Figure 1. The combined dataset of 5.8S, SSU, and LSU had an aligned length of 2,270 characters, and phylogenetic inference was estimated using both Maximum Likelihood and Bayesian Inference criteria. The isolate producing **1**, 067bN1.2, was placed on its own branch within the Lulworthiaceae, forming a sister clade to the clade including *Halazoon fuscus*, *Lulworthia medusa*, *Lulworthia* cf. *purpurea* and *Halazoon melhae*. Sequences of *Koralionastes ellipticus* were included to exclude the possibility that the isolate 067bN1.2 is part of the family Koralionastetaceae. *Koralionastes ellipticus* was placed outside of Lulworthiaceae.

### Isolation and Structure Elucidation

Compound **1** was selected for isolation due to its antibacterial activity in an initial screen of fractions from several understudied marine fungi. Compound **1** was the dominating peak in the active fraction from fungal isolate 067bN1.2 Lulworthiaceae sp., and subsequently the fungus was re-cultivated, cultures were extracted, and the compound was isolated using RP flash chromatography. The extraction of 1.4 L of fungal culture yielded 1,017.2 mg of extract.

**FIGURE 2:**
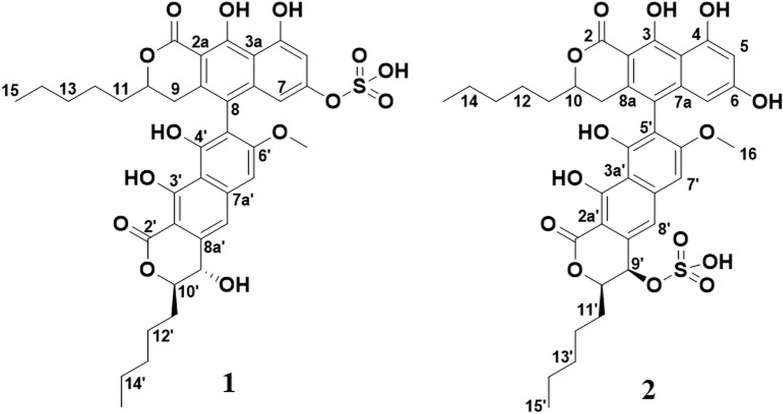
Structures of **1** and **2**.

Initially, attempts were made to isolate the compound using preparative HPLC. This strategy had several drawbacks, including unfavorable behavior of the compound in the preparative column. This resulted in the compound eluting over several minutes (band broadening) and carryover. A batch of the compound was, however, retrieved using this strategy, resulting in a compound later determined to be a structural isomer and artifact of compound **1** (referred to as **2** throughout this article), produced due to the acidic conditions in the mobile phase. The structures of **1** and **2** can be seen in Figure 2.

Flash chromatography was better suited for the isolation of **1**. This isolation strategy yielded 63.8 mg of **1**, corresponding to a yield of ∼45 mg/L culture medium. Compound **1** was obtained as a green colored substance. The molecular formula was calculated to be C_3__7_H_4__0_O_1__4_S by UHPLC-ESI-HRMS (*m/z* 741.2204 [M+H]^+^) (calculated as C_3__7_H_4__1_O_1__4_S, 741.2217), suggesting 18 degrees of unsaturation. The low-energy collision mass spectrum of **1** can be seen in Supplementary Figure 2. MS signals of a neutral loss of 80 Da (ESI+) was observed, indicating the presence of a sulfate group in the structure. The UV absorption maxima were 224, 260, and 373 nm, which corresponded well with the previously published dinapinones (Kawaguchi et al., 2013). The UV-vis spectrum for **1** can be seen in Supplementary Figure 3. The IR spectrum of **1** displayed absorption bands for sulfoxide (S=O, 1,002 cm^–1^), aromatic alkene (C=C, 1542 and 1,618 cm^–1^), carbonyl (C=O, 1,645 cm^–1^), alkane (C-H, 2,857 cm^–1^), aromatic alkene (C-H, 2926 cm^–1^), and hydroxyl (C-OH, 3455 cm^–1^) bonds. After isolation, the structure of **1** (Figure 2) was elucidated by 1D and 2D NMR experiments (Supplementary Figures 4–16).

Initial structure elucidation was made on the sample isolated by preparative HPLC with formic acid present in the mobile phases (compound **2**). The established molecular formula suggested a highly conjugated system. The purity of **2** was estimated to be ∼80% from a quantitative proton spectrum with respect to non-solvent impurities (Supplementary Figure 4). Four singlet protons were identified in the aromatic region, along with three O-CH signals at ∼4.5 ppm with complex couplings along with a methoxy singlet at 3.77 ppm. Furthermore, five hydroxyl protons were identified; three between 9.5 and 10.0 ppm, and two between 13.5 and 14.0 ppm. The deshielded nature of the latter sets them apart from the other hydroxyls and suggests they may be involved in an angled intramolecular hydrogen bond, which is commonly seen for keto-enol pair configurations such as this. All 37 carbons could be identified by 1D ^13^C NMR (Supplementary Figure 5), which showed **2** to contain a large number of aromatic quaternary carbons, two ester-like carbonyls, along with 10 peaks in the aliphatic region (Table 1).

**TABLE 1:** Summary of chemical shift and correlations for **2** (DMSO-*d*_6_).

Position	δ^13^C, type	δ^1^H, splitting (Hz)	COSY	HMBC (^1^H → ^13^C)
2	171.6, C	–	–	–
2’	171.0, C	–	–	–
2a’	99.4, C	–	–	–
2a	99.2, C	–	–	–
3	162.5, C	–	–	–
3’	161.5, C	–	–	–
3a’	108.6, C	–	–	–
3a	107.5, C	–	–	–
4	159.0, C	–	–	–
4’	154.9, C	–	–	–
5’	111.7, C	–	–	–
5	102.1, CH	6.35, s	–	3, 3a, 4, 6, 7
6	161.2, C	–	–	–
6’	160.7, C	–	–	–
7a	140.6, C	–	–	–
7a’	140.1, C	–	–	–
7	100.7, CH	6.04, s	–	3, 3a, 5, 6, 8
7’	99.7, CH	7.14, s	–	3’, 3a’, 4’, 5’, 6’, 8, 8’
8a’	137.4, C	–	–	–
8a	132.9, C	–	–	–
8	118.7, C	–	–	–
8’	117.5, CH	7.36, s	–	2’, 3a’, 4’, 6’, 7’a, 7’, 9’
9’	65.3, CH	4.69, d (*J* = 2.0)	–	2a’, 8’, 8a’, 10’, 11’
9	31.0, CH2	2.59, m	10	2a, 7a, 8, 8a, 10, 11
10’	83.2, CH	4.62, ddd (*J* = 7.9, 6.0, 2.0)	11’	2’, 8a’, 9’, 11’, 12’
10	79.4, CH	4.56, dddd (*J* = 9.6, 7.4, 5.5, 4.1)	9, 11	2, 8a, 12
11	34.2, CH2	1.59, dd (*J* = 16.7, 9.5) 1.68, dd (*J* = 16.5, 4.0)	10, 12	10, 12
11’	30.0, CH2	1.85, m	10’, 12’	9’, 10’, 12’, 13’
12’	24.7, CH2	1.48, 1.52, m	11’, 13’	11’, 13’, 14’
12	24.5, CH2	1.27, 1.36, m	11, 13	11, 13, 14
13’	31.3, CH2	1.23, m	12’, 14’	11’, 12’, 14’, 15’
13	31.6, CH2	1.36, m	12, 14	11, 12, 14, 15
14’	22.5, CH2	1.36, m	13’, 15’	12’, 13’, 15’
14	22.4, CH2	1.24, m	15	12, 13, 15
15’	14.4, CH3	0.92, m	14’	13’, 14’
15	14.3, CH3	0.82, m	14	13, 14
16	56.5, O-CH3	3.77, s	–	6’
OH3*	–	13.71, s		
OH3*	–	13.62, s		
OH4	–	9.80, s		
OH4’	–	9.51, s		
OH6	–	9.94, s		

*Ambiguous assignment.

**FIGURE 3:**
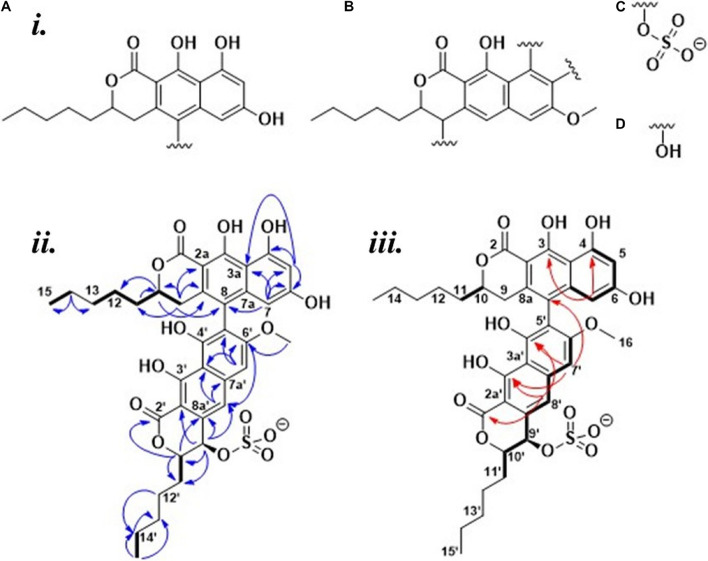
(*i*) Fragments identified for **2**: **(A,B)** The napthopyrone-like moieties; **(C)** a sulfate group; **(D)** a spare hydroxyl group. (*ii*) Elucidated structure of **2**: Bold bonds = COSY, blue arrows = HMBC, and (*iii*) red arrows = weak ^4^*J*_*CH*_ correlations, Bold bonds = 1,1-ADEQUATE.

HSQC, HMBC, and 1,1-ADEQUATE spectra (Supplementary Figures 6, 7) allowed the identification of two substituted napthopyrone-like moieties, as well as two five-membered aliphatic chains (denoted *C15-C11* and *C15’-C11’*, respectively), which were fully assigned using a combination of HSQC-TOCSY, TOCSY, COSY, and HMBC (Figure 3i). The aliphatic chains were determined to be attached at the *C10* position of the napthopyrone-like moieties by tracing the spin system into *H9* and *H9’*, respectively, and supported by multiple long-range ^1^H-^13^C correlations. The *C2* and *C2’* carbonyls could be directly assigned from long-range couplings from the *10*/*10’* position, but the hydroxyl carrying carbons in positions *3/3’* and *4/4’* could only be assigned through weak ^4^*J*_*CH*_ correlations from the aromatic protons (Figure 3iii).

The *OH-4* and *OH-6* could be assigned based on NOE correlations between *OH-6* and both *H5* and *H7*, while *OH-4* only displayed correlations with *H5*. The *OH-3* and *OH-3’* are predicted to have more deshielded chemical shifts due to their proximity to the carbonyl moiety and a probable intramolecular hydrogen bond—however, it was not possible to individually distinguish *OH3* and *OH-3’* due to the absence of any correlations in NOESY, ROESY, and HMBC spectra. Thus, four fragments could initially be elucidated (Figure 3i). A weak ^4^*J*_*C*__8H__7__’_ correlation could be detected, linking fragment ***A*** to fragment ***B*** (Figure 3i) at the *C8* and *C5’* positions, respectively, and thus the only remaining ambiguity is the position of the -SO^3–^ group vis-à-vis the remaining -OH in the 9’ or 4’ positions. The absence of NOEs and COSY correlations between *OH-4’* and *H9’* suggests that it is positioned at *C4’* with the sulfate positioned at *C9’* (Figure 3ii). The ^3^*J*_*HH*_ coupling constant between *H9*’ and *H10*’ was measured to be 2.0 Hz from line shape fitting the splitting of H9’, indicating that these protons are at a significantly offset dihedral angle to one another—thus suggesting a relative R/S or S/R configuration of 9’ and 10’. ^13^C prediction was consistent with the structure of **2** (Supplementary Figure 9), with a mean error of 2.79 ppm between the observed and predicted ^13^C shifts.

A second isolation where no acidic conditions were used, yielding **1**, was also examined. ^1^H NMR revealed significantly perturbed chemical shifts as well as line broadening and heterogeneity throughout the spectra (Supplementary Figure 11). Multiple resonances in the carbon spectrum (Supplementary Figures 12, 13), especially for two resonances in the carbonyl area (presumably C3 and C3’), are heterogenous, reflecting the nuclei existing in several stable, but slightly different micro environments. The same observation is made in the proton spectrum (Supplementary Figure 11) for H9’, OMe-6’, H5, H7, 4’-OH, and 4-OH. A major difference was observed in the non-acidic preparation (**1**), compared to **2**, the presence of a 9’-OH. At ∼15 ppm, two heterogeneous OH protons were observed, deshielded by approximately 1 ppm compared to the OH-3’s in the original sample preparation, while the three hydroxyls at ∼10 ppm could no longer be detected (Supplementary Figures 8–13). Thus, the detectable aromatic hydroxyl groups, identified as OH-4’ and OH-4, appeared to be involved in (stronger) hydrogen bonding, while three aromatic hydroxyls, the remaining OH-6, OH-3’ and OH-3, were unaccounted for. At the same time, the majority of all other nuclei in the molecule are shielded by approximately 0.5 ppm. Together, these observations suggest that the neutral pH preparation resulted in a different molecule, **1**, that formed loose aggregates in DMSO and methanol, stabilized by both hydrogen bonding (deshielding) and stacking (shielding) interactions. Overall, worse spectral quality resulted in that the C2 and C3 from **2** could not be individually assigned in **1**, although they must correspond to the two chemical shifts of 169.4 and 173 ppm by the logic of elimination. A number of the carbons show heterogenic peaks (notably the presumed C3 and C3’), most likely as the result of through space proximity to the sulfate group and sensitivity to its different possible conformation (details in section “Discussion”).

The identity of **1** was established to be identical to **2** with the only difference being that the sulfate group was attached to C6 instead of C9’, supported by the loss of the OH correlating with H5 and H7, and the appearance of an OH correlating with H9’ through a ^3^*J*_*HH*_. There is furthermore a heterogeneity and chemical shift perturbation hotspot (vis-à-vis **2**) around the C6 position to support the assignment of a C6 sulfate. All chemical shifts and correlations are summarized in Supplementary Table 3. The data do not unambiguously prove whether the 3-OH’s are deprotonated or if the signal is lost due to rapid exchange, but the fact that the OH-9’ is observable under the same conditions is an indicium for the OH-3’s to be deprotonated in **1**. No plausible resonance structures to explain the deprotonation and deshielding that does not involve the oxidation, and thus change in mass, have been found.

The non-aggregated **2** could be scavenged by lowering the pH of **1** with the addition of hydrochloric acid, upon which ^1^H and HSQC spectra of the two samples of **2** show a great resemblance (Supplementary Figure 10). The molecular formula of **2** and **1** as well as the scavenged **2** were identical in the two preparations, as no change in mass was observed by high-resolution mass spectrometry.

### Antibacterial Activity Against Reference and Clinical Strains

Compound **1** was tested against six reference bacteria (four Gram-positive and two Gram-negative strains). The compound was active against two of the Gram-positive reference strains, *S. aureus* and *S. agalactiae*, with MIC values of 6.25 and 12.5 μg/ml, respectively. No activity was observed against the Gram-negative strains, *E. coli* and *P. aeruginosa*, or the Gram-positive *E. faecalis* or MRSA strain (Supplementary Table 4). As bacterial resistance toward available antibiotics is the main challenge in future treatment of pathogenic diseases, **1** was tested against a panel of drug-resistant clinical strains (Supplementary Table 2). The panel included five MRSA and six VRE strains. Compound **1** was also tested in a pre-screen against four Gram-negative clinical bacterial strains: *E. coli*, *Klebsiella pneumoniae*, *Acinetobacter baumanii*, and *P. aeruginosa* (all ESBL-Carba). No activity was detected against the Gram-negative bacteria (Supplementary Table 4). Compound **1** showed activity against the MRSA strains with MICs in the 1.56–6.25 μg/ml (2.12–8.44 μM) range, see Table 2. The activity of the compound was significantly less profound against the VRE strains (MIC = 50 μg/ml or higher) (Supplementary Table 4).

**TABLE 2:** Minimal inhibitory concentrations (MICs) of **1** against reference strains and clinical isolates.

Strain type	Strain	MIC in μg/ml
*Clinical strains*	*S. aureus* N315	1.56
	*S. aureus* 85/2082	3.13
	*S. aureus* NCTC 10442	3.13
	*S. aureus* WIS [WBG8318]	6.25
	*S. aureus* IHT 99040	3.13
*Reference strains*	*S. aureus* ATCC^®^ 25923	6.25
	*S. agalactiae* ATCC^®^ 12386	12.5

*The median MIC values are reported (n = 12 for clinical isolates, n = 9 for reference strains).*

To investigate if **1** has bacteriostatic or bacteriocidal effects on the two reference strains *S. aureus* and *S. agalactiae*, both were incubated with the compound at 12.5 and 25 μg/ml overnight and subsequently plated onto agar. For *S. aureus*, there was no growth on the plates after overnight incubation, indicating a bacteriocidal effect of **1**. For *S. agalactiae*, one of the parallels at 12.5 μg/ml (MIC of **1** against this bacterium) displayed growth on the agar plate, which was expected as visual growth could also be seen in the microtiter plate for this parallel. The remaining five parallels at this concentration, and the concentration above, had no growth in the microtiter plates, or on agar after overnight incubation. This strongly indicates that **1** also has bacteriocidal effect on *S. agalactiae*. Compound **1** was also tested together with the efflux pump inhibitor reserpine to see if the lack of activity toward Gram-negative strains was caused by efflux of **1**, but no activity was obtained.

### Inhibition of Biofilm Production and Eradication of Established Biofilm

**FIGURE 4:**
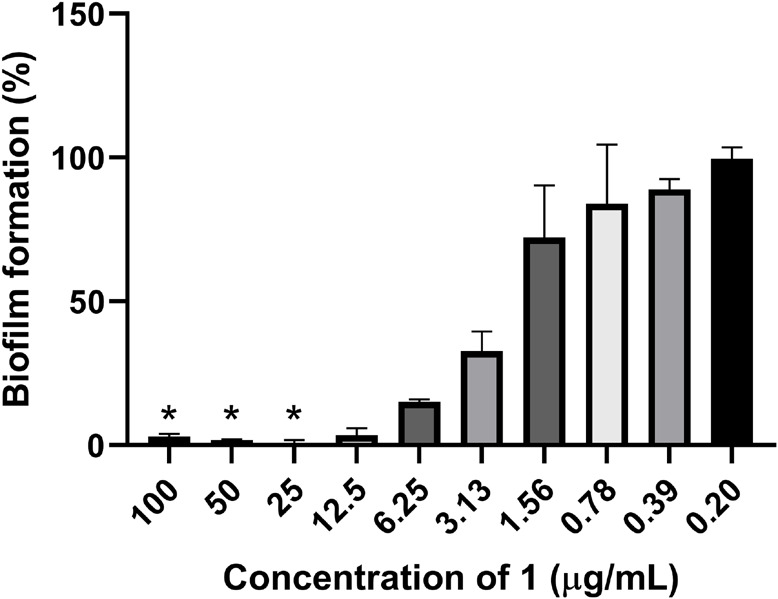
Inhibition of bacterial biofilm formation by **1** against the biofilm producing *S. epidermidis*. *The bacterial growth was completely inhibited at compound concentrations down to 25 μg/ml.

The ability of **1** to inhibit biofilm production by *S. epidermidis* and to remove established *S. epidermidis* biofilm was assessed. In the biofilm inhibition assay, the biofilm production was completely inhibited (below 5% biofilm formation) down to 12.5 μg/ml (Figure 4). Clear inhibition of the bacterial growth could also be observed to 25 μg/ml by visual inspection of plates before fixation of biofilm, raising the question if the biofilm inhibition is mainly caused by growth inhibition of the bacterium. To further evaluate the potential biofilm activity, removal of established biofilm was assessed. There was no activity of **1** at concentrations up to 100 μg/ml against the established biofilm, further supporting the hypothesis that the biofilm inhibition is mainly due to growth inhibition of the bacterium.

### Antiproliferative Activity Against Human Cells and Antifungal Activity

The antiproliferative activities of **1** was assessed against human melanoma cells (A2058), human non-malignant lung fibroblasts (MRC5), and human hepatocellular carcinoma cells (HepG2), in a concentration range of 6.25–100 μg/ml. The non-malignant cell line was included as a test for general toxicity, while the other cell line was included to assess possible anti-cancer activities. Antiproliferative activity was observed against all cell lines, with IC_50_ values of 15.5, 32, and 27 μg/ml against A2058, MRC5, and HepG2, respectively (Table 3). Compound **1** was also assayed for antifungal activity against *C. albicans* at concentrations up to 100 μg/ml, and no activity was seen.

**TABLE 3:** Antiproliferative activity (IC_50_) of **1** against human cell lines (*n* = 9).

Cell type	IC_50_ in μg/ml
A2058, melanoma	15.5 ± 0.6
MRC5, normal lung fibroblasts	32 ± 1
HepG2, hepatocellular carcinoma	27 ± 1

## Discussion

In this study, we describe the discovery, isolation, and characterization of the new secondary metabolite lulworthinone (**1**). This novel antibacterial compound was isolated from an extract of a slow-growing marine fungus of the family Lulworthiaceae. To the best of our knowledge, this is the first reported secondary metabolite isolated from this fungal family and the order Lulworthiales. Since the isolate did not branch close to the *Lulworthia* type species, *L. fucicola* (in the *Lulworthia sensu stricto* clade) and there was a lack of support at many nodes of the phylogenetic tree, we restrained from identifying the isolate 067bN1.2 to genus and determine its identity to family level only.

A fraction of the Lulworthiaceae sp. extract was nominated for chemical investigation as it was active in an initial antibacterial screen. The content of the active Lulworthiaceae sp. fraction was dominated by **1**, whose calculated elemental composition gave no hits in database searches, indicating that the compound suspected to be responsible for the observed antibacterial activity, was novel. In the attempt to utilize preparative HPLC to isolate this compound, **2** was generated during the procedure (acidic mobile phase). As compounds **1** and **2** have the same mass, HRMS analysis did not detect the change in the positioning of the sulfate group, and the sample from the preparative HPLC isolation was characterized using NMR, believing it was **1**. As preparative HPLC was deemed inconvenient for compound isolation, flash chromatography (neutral mobile phase) was utilized to isolate sufficient amounts of **1** to conduct a thorough characterization of the compound’s bioactivity. This method allows larger amounts of sample to be processed per run, but generally is less effective in separating compounds of interest from sample impurities, compared to preparative HPLC isolation. However, due to the high concentration of **1** in the extract, **1** was successfully isolated using this method. The resulting sample was submitted to NMR analysis to confirm its structure. The samples from both isolations were confirmed to be novel biarylic dimeric naphtho-α-pyrones substituted with a sulfate group. However, NMR analysis revealed that the sulfate group was located on different positions in the two compounds. The rearrangement was hypothesized to be catalyzed by the acidic nature of the HPLC mobile phase. This hypothesis was confirmed by subjecting **1** to acidic conditions (Supplementary Figure 10). The resulting sample was analyzed using NMR, confirming that **1** had indeed converted into **2**. As **2** was proven to be an artifact of **1**, all bioactivity testing was conducted using **1** isolated under neutral conditions.

**FIGURE 5:**
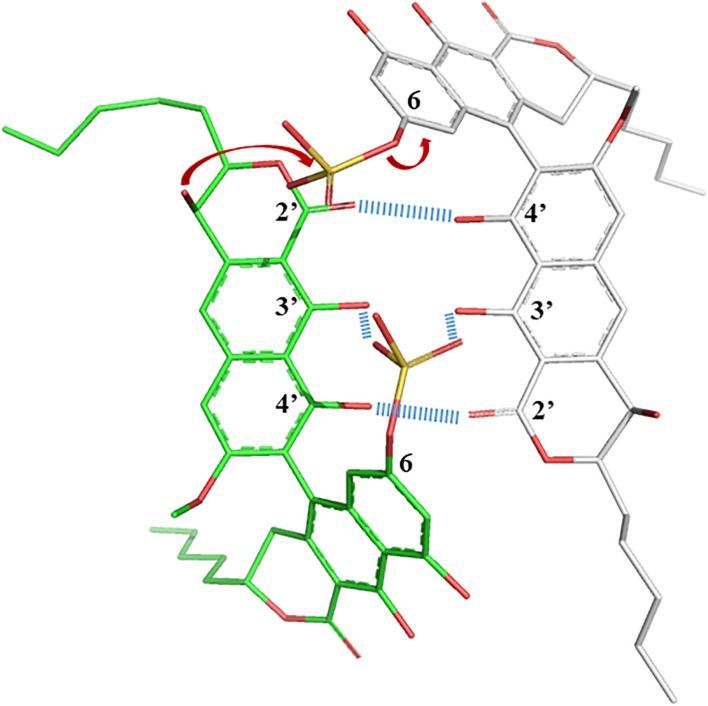
Crude sculpted and minimized structural model of **1** displaying the sulfate potential role in stabilizing oligomerization, as well as the possibility to intermolecularly react specifically at the C-9’ position to form **2** under acidic conditions.

The propensity of **1** to interact with itself to form higher-ordered structures, while **2** did not, offered some insight into their structural behavior in solution. In particular, the sulfate in the 6-position appeared to facilitate oligomeric aggregation, and a simple 3D model allows some speculation as to why this could be (Figure 5). The ground state of the naphthopyrone does not have the ability to form complementary “base pairs” with itself through hydrogen bonds between the carbonyls and hydroxyls. However, when the sulfate is in the 6-position, it can reach the C3 double OH “mismatch” in the three-dimensional structure and potentially stabilize the hydroxyls either by 4-coordnating a water molecule or a Na^+^ ion together with deprotonated 3’-hydroxyls, or by directly hydrogen bonding to the protonated hydroxyls. This would provide a feasible rationale for the propensity for aggregation of **1** but not of **2**. The structural dimer model also provides a plausible explanation as to why the sulfate group would specifically and irreversibly migrate to C9’ under acidic conditions even though the C9’ is expected to be a less likely position for the sulfate than any other phenol position. The sulfate is in an oligomeric state involving this kind of “base pairing” positioned to be intermolecularly attacked by the OH-9’ of the paired molecule, which is not possible in the monomeric state. Lowered pH is expected to ensure protonated sulfate, which would make it more susceptible for an electrophilic attack from OH-9’. If oligomeric states are indeed stabilized by the coordination of water or sodium, then lowered pH and the protonation of the 3- and 3’-oxygens would further destabilize the oligomer, which together with the lack of stabilization from the position 6 sulfate would make both the association and the reaction irreversible and trap the sulfate in the 9’ position of monomeric **2** with lowered ability to self-aggregate.

*Lulworthia* spp. fungi have spores with end chambers containing mucus, which helps in spore attachment to surfaces (Jones, 1994). It has been observed that in liquid culture of the isolate 067bN1.2, the fungus forms a gel-like mucus, having the ability to adhere to the bottom of the culture flasks. No spores are formed in culture, and it remains unclear whether the mucus formed under cultivation of 067bN1.2 has chemical resemblance to the mucus in end chambers of *Lulworthia* spp. spores, as it has not yet been characterized. The sheathing of mucoid by *L. medusa* has been reported in a publication from 1973, where the fungus was found and isolated from a piece of submerged pine and cultivated in bottles in media supplemented with artificial seawater (Davidson, 1973). Also in the current study, the fungus was found to adhere to the culture flask during cultivation in artificial seawater media. Davidson hypothesizes around the physiological and ecological implications of the mucoid, important in cation binding and transport, for the adhesion of other microorganisms, avoiding desiccation in intertidal regions or for the production of a matrix to concentrate exoenzymes (Davidson, 1973). Compound **1** is isolated in high yields from the fungal culture, but the ecological role of naphthopyrone-type compounds is largely unclear. The antibacterial activity of **1**, however, could indicate a protective role against pathogenic attacks, but the compound may have other types of bioactivities as well. It has been speculated that similar compounds (bis-naphthopyrones) from filamentous ascomycetes were produced to protect the fungus from predators (Xu et al., 2019). The study found that several animal predators, like woodlice, preferred feeding on fungi that had disrupted aurofusarin synthesis, and also that predation stimulated the production of aurofusarin in several *Fusarium* species (Xu et al., 2019). We have also observed marine mites feeding on fruitbody contents of Lulworthiales fungi. It is thus possible that in the natural habitat of these fungi, the naphthopyrones are produced as a means of protection.

Compound **1** was found to be a dimeric biarylic naphtho-α-pyrone substituted with a sulfate group. The naphthopyrone moiety is recurring in nature, as monomers, dimers, and trimers, and has been found from several natural sources, like plants and filamentous fungi. Naphthopyrones have also previously been isolated from organisms from the marine environment (Li et al., 2016). Compounds from this class have shown different bioactivities, among these the inhibition of triacylglycerol synthesis (Kawaguchi et al., 2013), inhibition of enzymatic activity (Zheng et al., 2007), protection against animal predators (Xu et al., 2019), antimalarial activities (Isaka et al., 2010), and antiproliferative activities (Isaka et al., 2010; Li et al., 2016). Several of these compounds have displayed antibacterial activities against Gram-positive bacteria (Suzuki et al., 1992; Wang et al., 2003; Zheng et al., 2007; Boudesocque-Delaye et al., 2015; Rivera-Chavez et al., 2019). Lu et al. (2014) defined three groups of bis-naphtho-γ-pyrones based on the diaryl bond connection between the monomers, the chaetochromin-, asperpyrone-, and nigerone-type bis-naphtho-γ-pyrones. Based on this categorization, **1** would be categorized as an asperpyrone-type bis-naphtho-α-pyrone, due to the relative placement of the oxygen atoms in the pyrone moieties. Compound **1** is substituted with a sulfate group. One of the most abundant elements in seawater is sulfur, and many sulfated compounds have been isolated from marine organisms, mostly from marine invertebrates, but also from microorganisms (Kornprobst et al., 1998; Francisca et al., 2018). Compound **1** represents, however, the first report of a dimeric naphtho-α-pyrone substituted with a sulfate group.

In the current study, **1** was broadly assessed for potential bioactivities: antibacterial activities against bacterial reference strains and clinical strains, antiproliferative activities toward a selection of human cell lines, both malignant and non-malignant, anti-fungal activity, inhibition of bacterial biofilm formation, and the eradication of established bacterial biofilm. Intriguingly, **1** showed activity against multidrug-resistant MRSA strains with MICs between 1.56 and 6.25 μg/ml (2.12–8.44 μM). In comparison, a natural product originally isolated from *Clitophilus scyphoides* (organism name at time of isolation: *Pleurotus mutilus*, Basidiomycota) pleuromutilin showed MICs in a similar range against selected reference strains (e.g., MIC = 0.66 μM against *S. aureus*, MIC = 2.64 μM against *K. pneumoniae*, and MIC = 21.13 μM against *B. subtilis*) while having significantly higher MIC values against other reference strains (e.g., MIC ≥ 100 μM against *P. aeruginosa*) (Kavanagh et al., 1951). An optimized analog of pleuromutilin, lefamulin (Xenleta^®^), was approved as an antibiotic drug by the US Food and Drug Administration in 2019. The herein reported MIC values thus place **1** in an activity segment, which makes it an interesting candidate for further development toward becoming a marketed antibiotic drug. In comparison to other antibacterial napthopyrones, **1** falls within the same MIC range with regard to activity toward Gram-positive bacteria. Two heterodimers, isolated from the tubers of *Pyrenacantha kaurabassana*, showed antibacterial activity against different strains of *S. aureus* with MICs in the range of 2.7–89.9 μM (Boudesocque-Delaye et al., 2015). In a recent paper from 2019, mycopyranone, a new binaphthopyranone, was isolated from the fermentation broth of *Phialemoniopsis*. The compound showed antibacterial activity against both *S. aureus* and a MRSA strain, with MICs of ≤8.7 μM against both strains (Rivera-Chavez et al., 2019). Possibly the most known naphthopyrone, viriditoxin showed MICs in the 4–8 μg/ml range against different *Staphylococcus* isolates (Wang et al., 2003).

Furthermore, the lack of activity against the Gram-negative reference and clinical strains shows the selectivity of **1** against Gram-positive bacteria. Yet, no activity or weak activity was observed against the clinical VRE isolates and the reference strain of *E. faecalis*, indicating that the activity is selective toward groups of Gram-positives, in this case *S. aureus* and *S. agalactiae*. Surprisingly, no activity was observed against the reference MRSA strain, and the reason behind this is not clear. No activity was observed for the combination of **1** and the efflux pump inhibitor reserpine, indicating that the lack of susceptibility by Gram-negatives is caused by another mechanism. In the antiproliferative activity assay, the most potent activity of **1** was observed against the melanoma cells (IC_50_ = 15.5 μg/ml). Against the non-malignant lung fibroblasts, which were included as a test for general toxicity, the compound had an IC_50_ of 32 μg/ml, which is more than five times higher than the highest MIC value against the multidrug-resistant MRSA. The concentrations where **1** did not display any toxic effect on the cells (∼100% cell survival) were 20, 12.5, and 15 μg/ml for MRC5, A2058, and HepG2, respectively. This indicates that there is little overlap between the concentration where **1** has antibacterial activity and the concentration where toxicity occurs against the human cells. This observed difference is a good starting point when entering structure optimization, as it indicates that production of non-toxic variants of **1** can be obtained.

We isolated 45 mg/L of **1** when the Lulworthiaceae sp. fungus was grown in liquid media supplemented with sea salts. This shows that slow-growing marine fungi *sensu stricto* can produce high yields of novel compounds for chemical characterization and screening for biological activities. Compound **1** was found to be a novel sulfated dimeric naphthopyrone, and showed potent growth inhibition of multidrug-resistant MRSA with MICs down to 1.56 μg/ml, which is much lower than the IC_50_ detected against the non-malignant cell line (32 μg/ml). This study demonstrates that the family Lulworthiaceae and order Lulworthiales have biosynthetic potential to produce bioactive secondary metabolites and supports the view of Overy et al. (2014) that marine fungi *sensu stricto* should be studied for natural product discovery, despite their slow growth (Overy et al., 2014). Our study highlights the potential role of marine fungi *sensu stricto* in tackling the worldwide AMR crisis.

## Data Availability Statement

The datasets presented in this study can be found in online repositories. The names of the repository/repositories and accession number(s) can be found in the article/Supplementary Material.

## Author Contributions

MJ was responsible for conducting experiments, data analysis, and writing and revising the draft manuscript. PR and JI were responsible for the NMR analysis of the compound and the writing related to this. EJ conducted the antibacterial testing against the clinical bacterial isolates and wrote this section in the “Materials and Methods,” and contributed to the writing of the MIC results. KH assisted in writing and revision of the manuscript and contributed to the experiment design. TR did the initial isolation of the fungus and the phylogenetic analysis, contributed to the experiment design by selecting this fungus for the study, and revised the manuscript. JA and EH contributed to the conceptualization of the work, supervised the work, and revised the manuscript. All authors reviewed and approved the final manuscript.

## Conflict of Interest

The authors declare that the research was conducted in the absence of any commercial or financial relationships that could be construed as a potential conflict of interest.

## Publisher’s Note

All claims expressed in this article are solely those of the authors and do not necessarily represent those of their affiliated organizations, or those of the publisher, the editors and the reviewers. Any product that may be evaluated in this article, or claim that may be made by its manufacturer, is not guaranteed or endorsed by the publisher.
