# Supplementary material for: Lulworthinone, a New Dimeric Naphthopyrone From a Marine Fungus in the Family Lulworthiaceae With Antibacterial Activity Against Clinical Methicillin-Resistant Staphylococcus aureus Isolates
Source: Front Microbiol. 2021 Oct 1;12:730740. doi: 10.3389/fmicb.2021.730740 (PMC8517231; doi:10.3389/fmicb.2021.730740)
Supplement: Supplementary file 1 [file Data_Sheet_1.docx]

Supplementary Material

# Table of Content

**Supplementary Table 1:**Dataset of nrITS, nrLSU and nrSSU used for phylogenetic analysis of 067bN1.2. All sequences were acquired from Genbank.

**Supplementary Table 2:** Information regarding the clinical isolates used for antibacterial activity testing of **1**.

**Supplementary Table 3:** Summary of chemical shift and correlations for **1**.

**Supplementary Table 4:**Results for the MIC determination of **1** against clinical isolates and reference strains (MIC of 50 µg/ml or higher/above highest tested concentration).

**Supplementary Figure 1:**MrBayes tree from the 5.8S, SSU and LSU analysis, showing the placement of 067bN1.2 within the family Lulworthiaceae.

**Supplementary Figure 2:**Low-collision energy mass spectrum of lulworthinone (**1**) in ESI+.

**Supplementary Figure 3:**UV-Vis spectrum of lulworthinone (**1**).

**Supplementary Figure 4:** 1D proton spectrum of **2**.

**Supplementary Figure 5:** 1D carbon spectrum of **2**.

**Supplementary Figure 6:** Superimposed HSQC and HMBC of **2**.

**Supplementary Figure 7:** 1,1-ADEQUATE of **2**.

**Supplementary Figure 8:** ROESY (300 ms mixing time) of **2**.

**Supplementary Figure 9:**Predicted vs observed ^13^C chemical shift comparison.

**Supplementary Figure 10:**The HSQC peaks of the aromatic region of the second preparation of **1**, compared to the initial preparation of **2** in the presence of formic acid.

**Supplementary Figure 11:**Proton spectrum of **1** in DMSO-d_6_.

**Supplementary Figure 12:** Carbon spectrum of **1** in DMSO-d_6_.

**Supplementary Figure 13:**Expansion of the carbonyl/deep aromatic region of the carbon spectrum in Supplementary Figure 12.

**Supplementary Figure 14:**Superimposed HSQC (red/blue) and HMBC (black) of **1** in DMSO-d_6_.

**Supplementary Figure 15:**HMQC optimized for 4 Hz ^n^J_CH_ displays some of the important ^4^J_CH_ for assignment.

**Supplementary Figure 16:** NOESY (600 ms mixing time) of **1**.

**Supplementary Table 1:**Dataset of nrITS, nrLSU and nrSSU used for phylogenetic analysis of 067bN1.2. All sequences were acquired from Genbank.

| **Species** | **Strain** | **Source** | **nrITS** | **nrLSU** | **nrSSU** |
| --- | --- | --- | --- | --- | --- |
| *Achroceratosphaeria potamia* | JF 08139 | Submerged wood  of *Platanus* sp. | - | GQ996538 | GQ996541 |
| *Bimuria novae-zelandiae* | CBS 107.79 | Soil | - | AY016356 | AY016338 |
| *Cumulospora marina* | MF46 | Submerged wood | - | GU252135 | GU252136 |
| *Cumulospora varia* | GR78 | Submerged wood | - | EU848578 | EU848593 |
| *Halazoon fuscus* | NBRC 105256 | Driftwood | - | GU252147 | GU252148 |
| *Halazoon melhae* | MF819 | Drift stems of  *Phragmites*  *australis* | - | GU252143 | GU252144 |
| *Hydea pygmea* | NBRC 33069 | Driftwood | - | GU252133 | GU252134 |
| *Kohlmeyeriella crassa* | NBRC 32133 | Sea foam | LC146741 | LC146742 | AY879005 |
| *Kohlmeyeriella tubulata* | PP115 | Marine environment | - | AF491265 | AY878998 |
| *Koralionastes ellipticus* | JF08139 | Coral rocks with sponges | - | EU863585 | EU863581 |
| *Letendraea helminthicola* | CBS 884.85 | Yerba mate | EU715680 | AY016362 | AY016345 |
| *Lindra marinera* | JK 5091A | Marine environment | - | AY878958 | AY879000 |
| *Lindra obtusa* | NBRC 31317 | Sea foam | LC146744 | AY878960 | AY879002 |
| *Lindra thalassiae* | AFTOL 413 | Marine environment | DQ491508 | DQ470947 | DQ470994 |
| *Lulworthia atlantica* | FCUL210208SP4 | Sea water | KT347205 | JN886843 | KT347193 |
| *Lulworthia* cf*. opaca* | CBS 21860 | Driftwood in  seawater | - | AY878961 | AY879003 |
| *Lulworthia* cf*. purpurea* | FCUL170907CP5 | Sea water | KT347219 | JN886824 | KT347201 |
| *Lulworthia fucicola* | ATCC 64288 | Intertidal wood | - | AY878965 | AY879007 |
| *Lulworthia grandispora* | NTOU3841 | Driftwood | - | KY026048 | KY026044 |
| *Lulworthia lignoarenaria* | AFTOL 5013 | Marine environment | - | FJ176903 | FJ176848 |
| *Lulworthia medusa* | JK 5581 | Spartina | - | AF195637 | AF195636 |
| Lulworthiaceae | 067bN1.2 | Driftwood | MW377595 | MW375591 | MW375590 |
| *Matsusporium tropicale* | NBRC 32499 | Submerged wood | - | GU252141 | GU252142 |
| *Moleospora maritima* | MF836 | Drift stems of  *Phragmites*  *australis* | - | GU252137 | GU252138 |
| *Paralulworthia gigaspora* | MUT 435 | *P. oceanica* –  rhizomes | MN649242 | MN649250 | MN649246 |
| *Paralulworthia posidoniae* | MUT 5261 | *P. oceanica* –  rhizomes | MN649245 | MN649253 | MN649249 |
| *Setosphaeria monoceras* | CBS 154.26 | n.d. | DQ337380 | AY016368 | DQ238603 |
| *Zalerion maritima* | FCUL280207CP1 | Sea water | KT347216 | JN886806 | KT347203 |

**Supplementary Table 2:** Information regarding the clinical isolates used for antibacterial activity testing of **1**.

| **Clinical isolate** | **Antibiotic Resistance Mechanism** | **Reference** | **Source (gifted/bought)** |
| --- | --- | --- | --- |
| *S. aureus* N315 | MRSA | Ito et al. (1999). Cloning and nucleotide sequence determination of the entire mec DNA of pre-methicillin-resistant *Staphylococcus aureus* N315. *Antimicrob. Agents Chemother,43*, 1449-1458. doi: 10.1128/AAC.43.6.1449 | T. Ito, Juntendo University, Tokyo (Japan) |
| *S. aureus* 85/2082 |  | Suzuki et al. (1993). Distribution of mec Regulator Genes in Methicillin-Resistant  *Staphylococcus* Clinical Strains. *Antimicrob. Agents Chemother.*,*37*, 1219-1226. doi: 0066-4804/93/061219-08$02.00/0 | T. Ito, Juntendo University, Tokyo (Japan) |
| *S. aureus* NCTC 10442 |  | Ito et al. (2001).Structural comparison of three types of staphylococcal cassette chromosome mec integrated in the chromosome in methicillin-resistant *Staphylococcus aureus*.  *Antimicrob. Agents Chemother,45*, 1323-1336. doi: 10.1128/AAC.45.5.1323-1336.2001. | NCTC |
| *S. aureus* WIS [WBG8318] |  | Ito et al. (2004).Novel Type V Staphylococcal Cassette Chromosome mec Driven by a Novel Cassette Chromosome Recombinase, ccrC. *Antimicrob. Agents. Chemother.,48*, 2637–2651. doi: 10.1128/AAC.48.7.2637-2651.2004 | K. Hiramatsu, Juntendo University, Tokyo, (Japan) |
| *S. aureus* IHT 99040 |  | Salmenlinna, S., Lyytikäinen, O., & Vuopio-Varkila, J. (2002).Community-Acquired Methicillin-Resistant *Staphylococcus aureus*, Finland.*Emerging infectious diseases*, *8*, 602–607.doi: 10.3201/eid0806.010313 | Saara Salmenlinna (IHT, Helsinki, Finland) |
| *E. faecium* 50673722 | VRE | Sivertsen A, Janice J, Pedersen T,Wagner TM, Hegstad J, Hegstad K. 2018. Theenterococcus cassette chromosome, agenomic variation enabler in enterococci.*mSphere*, 3, 1-13. doi:10.1128/mSphere.00402-18 | K-res ^a^ |
| *E. faecium*50901530 |  | - | K-res ^a^ |
| *E. faecium*K36-18 |  | - | K-res ^a^ |
| *E. faecium*50758899 |  | - | K-res ^a^ |
| *E. faecium*TUH50-22 |  | - | K-res ^a^ |
| *E. faecium*1-H-4 |  | - | K-res ^a^ |
| *E. coli* 50676002 | ESBL-Carba | - | K-res ^a^ |
| *K. pneumoniae* K47-25 |  | - | K-res ^a^ |
| *A. baumanii* K47-42 |  | - | K-res ^a^ |
| *P. aeruginosa* K34-7 |  | - | K-res ^a^ |
| *E.coli* ATCC 25922 | - | ATCC | ATCC |

^a^ 2006-2015 The Norwegian National Advisory Unit on Detection of Antimicrobial Resistance (K-res), University Hospital of North Norway – UNN.

**Supplementary Table 3:** Summary of chemical shift and correlations for **1**(DMSO-*d*_6_).

| **Position** | **δ^13^C, type** | **δ^1^H, splitting (Hz)** | **COSY** | **HMBC (^1^H → ^13^C)** |
| --- | --- | --- | --- | --- |
| 2 | 170.0*,C | - | - | - |
| 2' | 169.7, C | - | - | - |
| 2a' | 98.3, C | - | - | - |
| 2a | 98.0, C | - | - | - |
| 3 | 173.8*, C | - | - | - |
| 3' | 173.8, C | - | - | - |
| 3a' | 112.2, C | - | - | - |
| 3a | 113.0, C | - | - | - |
| 4 | 162.0, C | - | - | - |
| 4' | 160.3, C | - | - | - |
| 5' | 108.7, C | - | - | - |
| 5 | 101.8, CH | 6.55, h | - | 3a, 4, 6, 7 |
| 6 | 155.3, C | - | - | - |
| 6' | 160.0, C | - | - | - |
| 7a | 138.2, C | - | - | - |
| 7a' | 139.3, C | - | - | - |
| 7 | 104.6, CH | 6.05, h | - | 3a, 5, 6, 8 |
| 7' | 96.6, CH | 6.69, h | - | 3',3a', 5', 6', 8' |
| 8a' | 139.2, C | - | - | - |
| 8a | 133.3, C | - | - | - |
| 8 | 113.2, C | - | - | - |
| 8' | 110.9, CH | 6.74, h | - | 2',2a',3’,3a',7a',7',8a',9' |
| 9' | 65.7, CH | 4.69, h | OH9' | 2a', 8', 8a', 10' |
| 9 | 31.7, CH2 | 2.40/2.57, m | 10 | 8, 8a, 10 |
| 10' | 80.3, CH | 4.62, m | 11' | 8a', 9', 11', 12' |
| 10 | 77.2, CH | 4.56, m | 9 | 8a, 12 |
| 11 | 33.7, CH2 | 1.52, m  1.64, m | 12 | 10 |
| 11' | 29.5, CH2 | 1.78, m | 10', 12' | 10' |
| 12' | 24.2, CH2 | 1.27/1.34, m | 11',13' | 11',13',14' |
| 12 | 24.3, CH2 | 1.47, m | 11, 13 | 11,13,14 |
| 13' | 31.2, CH2 | 1.34, m | 12' | 14',15' |
| 13 | 30.9, CH2 | 1.21, m | 12 | 14,15 |
| 14' | 22.1, CH2 | 1.34, m | 15' | 13',15' |
| 14 | 22.0, CH2 | 1.23, m | 15 | 13,15 |
| 15' | 14.0, CH3 | 0.90, t (J=6.5) | 14' | 13',14' |
| 15 | 13.9, CH3 | 0.81, h | 14 | 13,14 |
| 16 | 55.4, O-CH3 | 3.77, h | - | 6' |
| OH3^*^ | - | -, s |  |  |
| OH3^*^ | - | -, s |  |  |
| OH4 | - | 14.74, h |  | 4,3a,5 |
| OH4’ | - | 14.65, h |  | 4',3a',5 |
| OH9’ | - | 5.51, h | 9' |  |

*Ambiguous assignment

**Supplementary Table 4:**Results for the MIC determination of **1** against clinical isolates and reference strains (MIC of 50 µg/ml or higher/above highest tested concentration).

| **Strain type** | **Strain** | **MIC in µg/ml** |
| --- | --- | --- |
| *Clinical isolates* | *E. faecium*  50673722 | >100 |
|  | *E. faecium*  50901530 | >100 |
|  | *E. faecium*  K36-18 | 100 |
|  | *E. faecium*  50758899 | >100 |
|  | *E. faecium*  TUH50-22 | 100 |
|  | *E. faecium*  1-H-4 | 50 |
|  | *E. coli*  50676002 | >100 |
|  | *K. pneumoniae*  K47-25 | >100 |
|  | *A. baumanii*  K47-42 | >100 |
|  | *P. aeruginosa*  K34-7 | >100 |
| *Reference strains* | *Enterococcus faecalis*  ATCC® 29212 | >100 |
|  | Methicillin resistant *S. aureus*  ATCC® 33591 | >100 |
|  | *Escherichia coli*  ATCC® 25922 | >100 |
|  | *Pseudomonas aeruginosa*  ATCC® 27853 | >100 |


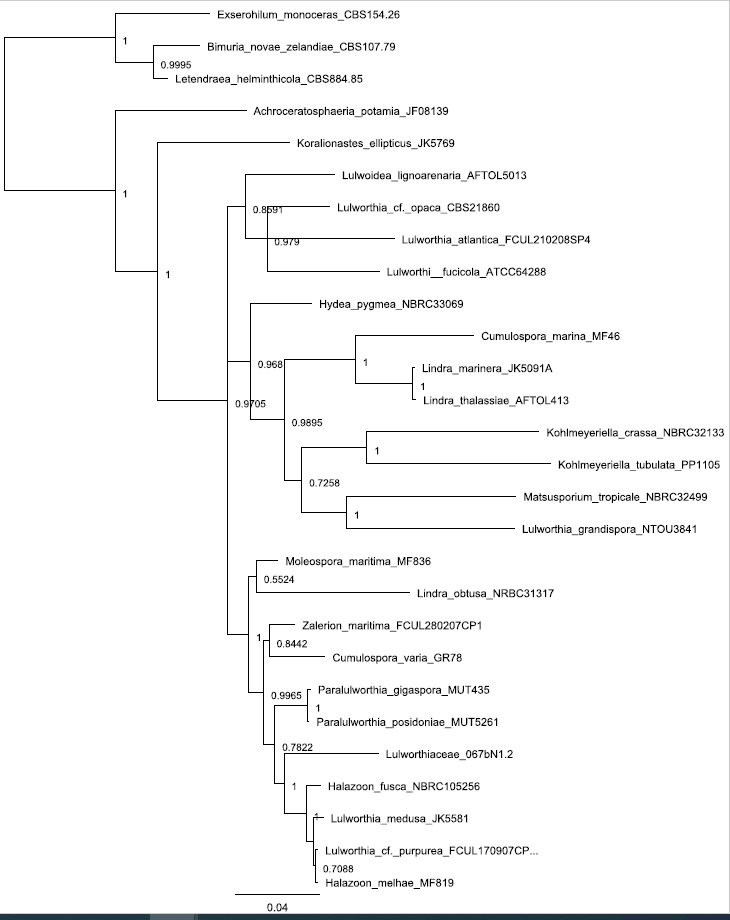


**Supplementary Figure 1:**MrBayes tree from the 5.8S, SSU and LSU analysis, showing the placement of 067bN1.2 within the family Lulworthiaceae. Node support given as posterior probabilites. *Exserophilum monoceras, Letendraea helminthicola, Bimufia novae-zelandiae* and *Achroceratosphaeria potamia* were included as outgroups taxa. *Koralionastes ellipticus* was included as a member of the family Koralionastetaceae. The remaining sequences are all part of Lulworthiaceae.


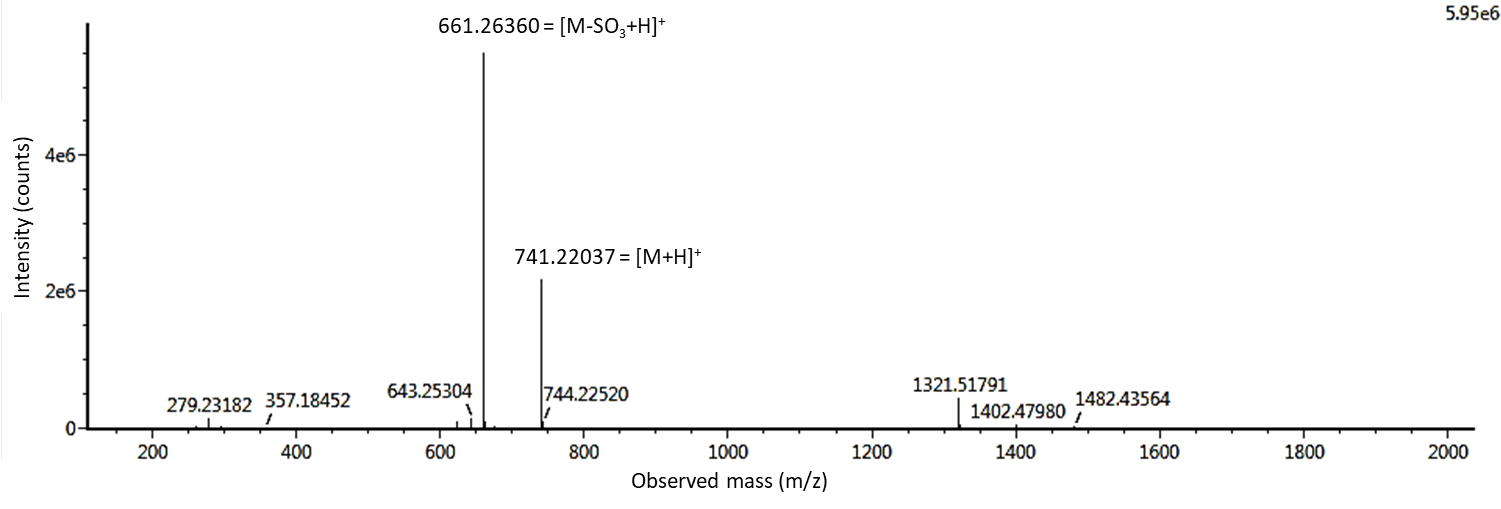


**Supplementary Figure 2:**Low-collision energy mass spectrum of lulworthinone (**1**) in ESI+.


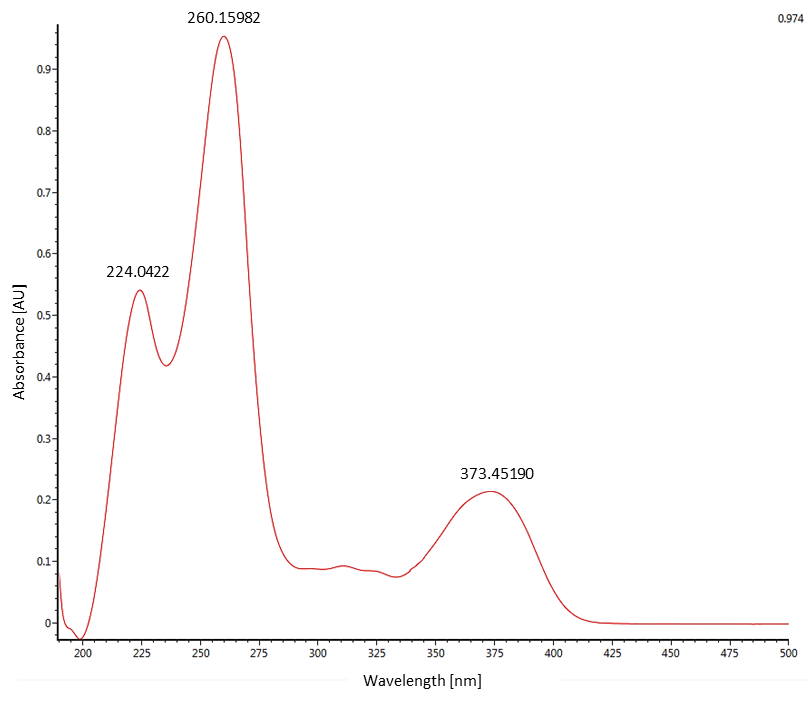


**Supplementary Figure 3:**UV-Vis spectrum of lulworthinone (**1**).

**Supplementary Figure 4:** 1D proton spectrum of **2**.

**Supplementary Figure 5:** 1D carbon spectrum of **2**.

**Supplementary Figure 6:** Superimposed HSQC and HMBC of **2**.

**Supplementary Figure 7:** 1,1-ADEQUATE of **2**.

**Supplementary Figure 8:** ROESY (300 ms mixing time) of **2**.

*
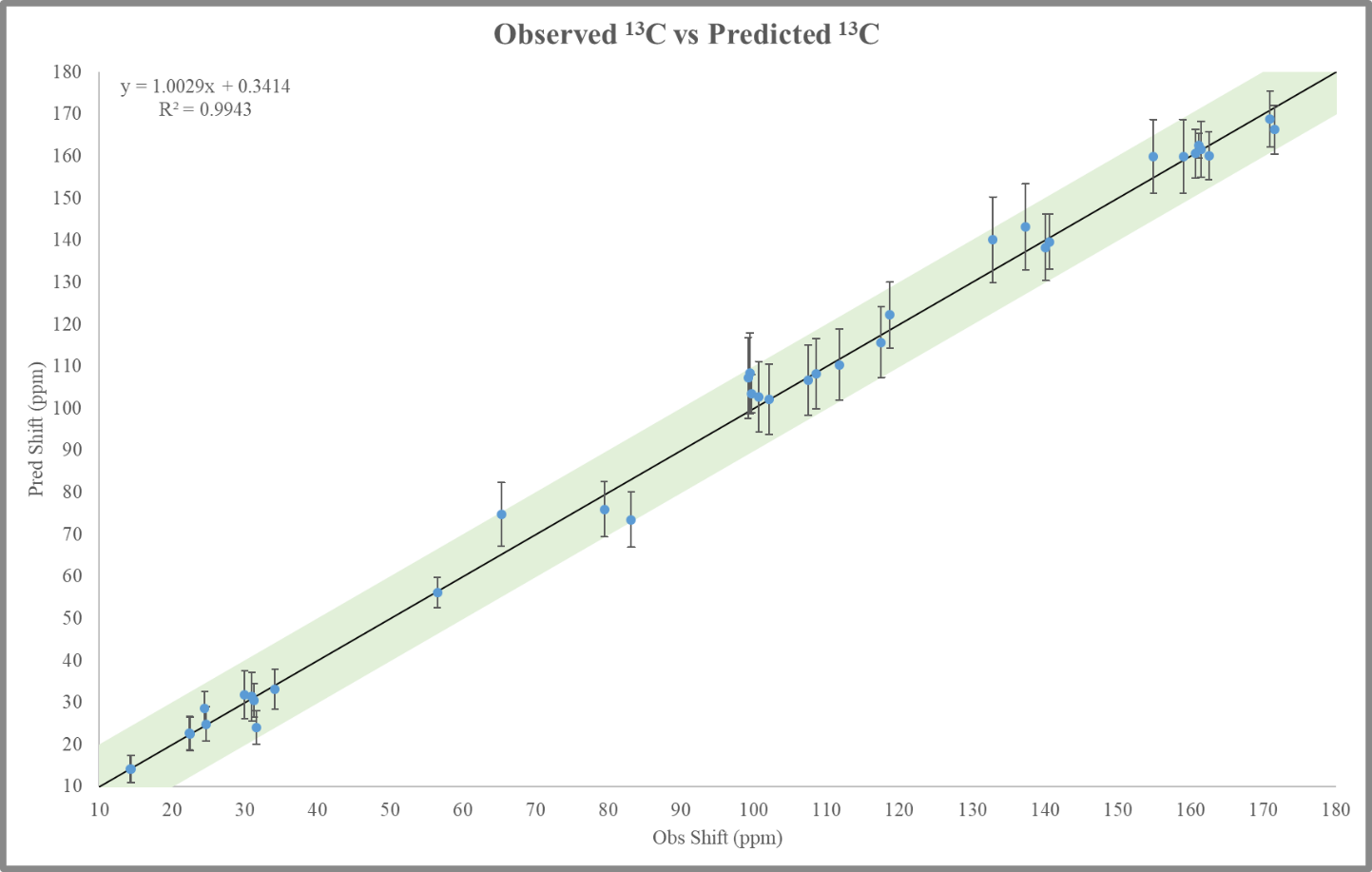
*

**Supplementary Figure 9:**Predicted vs observed ^13^C chemical shift comparison. Average error of 2.79 ppm, R2 of 0.9943. Green region is equivalent to an error of +/- 10 ppm, black line y = x. Errors for prediction given by MestreNova Modgraph desktop prediction.

**Supplementary Figure 10:**The HSQC peaks of the aromatic region of the second preparation of **1** (red) at neutral (top) and after addition of acid (bottom), compared to the initial preparation of **2** in the presence of formic acid (black).

**Supplementary Figure 11:**Proton spectrum of **1** in DMSO-d_6_.

**Supplementary Figure 12:** Carbon spectrum of **1** in DMSO-d_6_.

**Supplementary Figure 13:**Expansion of the carbonyl/deep aromatic region of the carbon spectrum in Supplementary Figure 12. Compared to **2**, **1** only has 4 carbons in the 160-165 range, and instead has 4 carbons in the 169-175 range. Integrals should be interpreted conservatively as it is ill advised to integrate carbon signals, but in this case we only qualitatively compare quaternary carbons to each other where the stead state noe enhancement is expected to be low and their T1 relaxation times are expected to be similarly slow. Without reading too much into it, it appears that C3 and C3’ are not hidden among the other carbons in the 160-165 range but have indeed shifted to the more deshielded region normally associated with carbonyl resonances.

**Supplementary Figure 14:**Superimposed HSQC (red/blue) and HMBC (black) of **1** in DMSO-d_6_.

**Supplementary Figure 15:**HMQC optimized for 4 Hz ^n^J_CH_ displays some of the important ^4^J_CH_ for assignment.

**Supplementary Figure 16:** NOESY (600 ms mixing time) of **1**. OH-4’ displays NOE correlations with both H7 and H9, showing that the two ring systems are either rotating quickly, exist in several conformations, or are offset relative to each other allowing one interaction on top of OH-4’ and the other below OH-4’.
